# Distinct immunophenotypes and prognostic factors in renal cell carcinoma with sarcomatoid differentiation: a systematic study of 19 immunohistochemical markers in 42 cases

**DOI:** 10.1186/s12885-017-3275-8

**Published:** 2017-04-27

**Authors:** Wenjuan Yu, Yuewei Wang, Yanxia Jiang, Wei Zhang, Yujun Li

**Affiliations:** 1 Department of Pathology, The Affiliated Hospital of Qingdao University, 16 Jiangsu Road, Qingdao, 266003 China; 2 Department of Pathology, 401 Hospital of People’s Liberation Army, 22 Minjiang Rd, Qingdao, 266071 China; 3 Department of Vascular Surgery, The Affiliated Hospital of Qingdao University, Qingdao, 266003 China

**Keywords:** Renal cell carcinoma, Sarcomatoid, Pathogenesis, Immunohistochemistry

## Abstract

**Background:**

Renal cell carcinoma (RCC) with sarcomatoid differentiation is a relatively rare tumor containing both carcinoma and sarcomatoid components. However, there has not been a systemic study on immunophenotypes of renal cell carcinoma with sarcomatoid differentiation, especially using some renal specific immunohistochemical markers. In this study, we aimed to comprehensively investigate the distinct immunophenotypes of RCC with sarcomatoid differentiation to analyze the pathogenesis of sarcomatoid differentiation and identify new prognostic factors in RCC with sarcomatoid differentiation.

**Methods:**

A total of 42 cases of RCCs with sarcomatoid differentiation were enrolled into the study. Immunohistochemistry study was performed on tissue microarrays to evaluate the expressions of 19 immunohistochemical markers including a series of epithelial, mesenchymal markers and RCC specific markers. Kaplan-Meier method was applied to assess the prognostic values of CD10, CAIX, p53 and Bcl-2.

**Results:**

Histologically, 42 cases of RCCs with sarcomatoid differentiation presented with different proportions of carcinoma and sarcomatoid components. The cohort contained 35 cases of clear cell renal cell carcinoma (CCRCC) and 7 cases of chromophobe renal cell carcinoma (ChRCC) based on the carcinoma components. Immunohistochemically, all cases were positive for vimentin, and 80% of cases showed immunostaining for at least one epithelial marker, such as CK, EMA, CK7 and CK18. Notably, the expression rates of CAIX, CD10 and PAX8 in sarcomatoid cells were 76%, 76% and 64%, respectively. The carcinoma component of the tumors showed differentient labeling for CAIX, CD10, vimentin, CK7 and CD117 in CCRCC vs ChRCC, but the sarcomatoid component lost the specificity for these markers ( *p* < 0.05). Patients with positive expressions of CAIX, p53 and Bcl-2 had a poor prognosis.

**Conclusions:**

The sarcomatoid cells in RCC with sarcomatoid differentiation express both epithelial and mesenchymal markers, supporting their epithelial origin. PAX8, CAIX and CD10 could be used as the reliable and useful markers to determine the renal origin of sarcomatoid cells such as in fine needle aspiration cases and metastatic RCC with sarcomatoid differentiation. CAIX, p53 and Bcl-2 might play important roles in the transformation from renal cell carcinoma to high malignant sarcomatoid differentiation, and these three immunohistochemical markers are adverse prognostic factors for the survival of patients with RCC with sarcomatoid differentiation.

## Background

Renal cell carcinoma (RCC) with sarcomatoid differentiation is a rare renal carcinoma, also called sarcomatiod renal cell carcinoma, spindle cell carcinoma and carcinomatous sarcoma, etc. One of its key histological features is the presence of both carcinoma and sarcomatoid differentiation in the tumor [1]. According to the 2004 and 2016 WHO Classification of Tumors of the Urinary System and Male Genital Organs, RCC with sarcomatoid differentiation is not recognized as a separate and distinct entity and the sarcomatoid component could arise from the genetic background of any of the RCC subtypes [2]. RCC with sarcomatoid differentiation presents unique histological characteristics, invasive behavior and poor prognosis [3]. Due to the low morbidity and the deficiency of study data, the pathogenesis of the transformation from carcinoma to sarcomatoid component along with the prognostic factors for RCC with sarcomatoid differentiation remains unelucidated. Herein we performed our immunohistological study using 19 immune markers, including the renal specific markers PAX8 and CAIX in 42 cases of RCCs with sarcomatoid differentiation. Our study was focused on the analysis of the expression differences between those two components. This study may shed some light on revealing the pathogenesis and assessing the prognosis of the patients with this relatively rare renal carcinoma.

## Methods

Among the 42 cases of RCCs with sarcomatoid differentiation, 35 cases were collected in the Affiliated Hospital of Qingdao University and 7 cases were collected in 401 Hospital of People’s Liberation Army from November 2003 to January 2015. All cases were re-diagnosed by two pathologists majored in Genitourinary Pathology. The samples were made into 2 tissue microarrays and there were 42 punches in each tissue microarray. Each tumor was made into 2 punches including carcinoma and sarcomatoid components separately based on the observation under microscope. The diameter of the cores was 2 mm.

All tissues were 4-um-thick, fixed with 4% neutral formaldehyde, and embedded in paraffin for H&E staining. Immunohistochemistry was performed on tissue microarrays comprising of 42 cases containing both typical renal cell carcinoma cells and sarcomatoid cells. Primary antibodies used in the study including vimentin (Cell Marque, V9), cytokeratin (CK) (Origene, AE1/AE3), epithelial membrane antigen (EMA) (Thermo, E29), cytokeratin-7 (CK7) (Cell Marque, OV-TL12/30), cytokeratin-18 (CK18) (Origene, UMAB50), high molecular weight cytokeratin (HMW-CK) (Cell Marque, CKHMW), and CAIX (Novus, polyclonal), CD10 (Ventana, SP67), PAX8 (Ventana, MRQ-50), alpha-methylacylCoA racemase (AMACR) (Zeta,13H4), CD117 (Ventana, 9.7), CD99 (Covance, O13), SMA (Origene, 1A4), CD34 (Leica, OBEnd/10), S100 (Thermo, 4c4.9), Melanoma (Cell Marque, HMB45), and Melan A (Cell Marque, A103), p53 (Ventana, DO-7), and Bcl-2 (Thermo, 8c8). UltraView™ DAB detection kit was purchased from Ventana (Arizona, America). All immunohistochemistry assays were performed on the Roche BenchMark XT fully automatic IHC/ISH instrument by optimized protocols. Positive and negative controls were used in this study.

The positive staining of vimentin, CK, CK7, CK18, HMW-CK, CD34, AMACR, SMA, Bcl-2, HMB45 and melan A predominantly appeared in the tumor cell cytoplasm, whereas p53 and PAX8 mainly appeared in the nuclei. CD99, EGFR and CAIX tended to express on the cell membrane. EMA, CD10 and CD117 expressed on the cell membrane or cytoplasm; S100 expressed in the cytoplasm and nuclei. The positive staining was first scored as 0, 1, 2, 3 based on the staining intensity (no staining, faint, mild, strong) and the percentage of positively stained cells was (0%, ≤25%, 26% ~ 75%, >75%), respectively. Two scores then multiplied together, which were considered as the final scores, grading as – (0–1), 1+ (2–4), 2+ (5–6), 3+ (7–9).

### Clinical follow-up and survival analysis

Follow-up data were collected for 33 cases. Survival was calculated from the date of surgery to the death or the last follow-up visit. For the analysis, only deaths for RCCs with sarcomatoid differentiation were considered as events. Survival curves were derived from Kaplan-Meier analysis and log-rank test to compare overall survival between different groups based on the expressions of CD10, CAIX, p53 and Bcl-2 since the four markers might play roles in the proliferation of tumors. Furthermore, in order to investigate the factors that may affect survival patterns, the *P* values for prognostic factors analyses were adjusted for multiple analyses using Cox regression model.

### Statistical analysis

SPSS 13.0 software was applied to perform statistical analysis. Statistical significance was tested by chi-square test (or Fisher’s exact test) to compare each marker’s expression in the carcinoma and sarcomatoid components. Statistical significance was considered as *P* value less than 0.05.

## Results

**Fig. 1:**
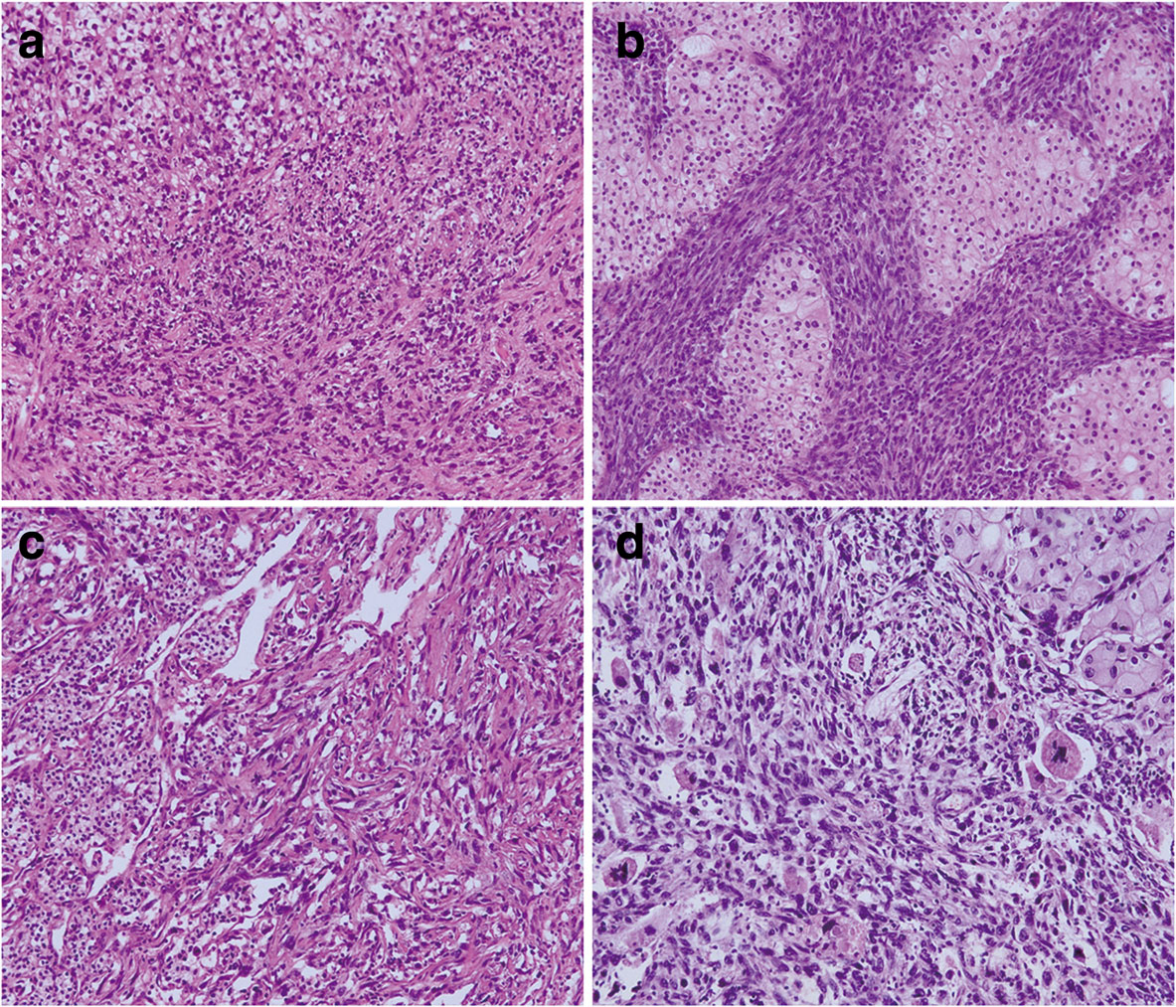
The histopathological figures of RCC with sarcomatoid differentiation. **a** CCRCC together with fibrosarcomatoid cells **b** ChRCC together with fibrosarcomatoid cells **c** Clear cell carcinoma together with leiomyosarcoma-like cells **d** ChRCC together with malignant fibrous histiocytoma-like cells. H&E × 100

There were 35 clear cell renal cell carcinomas (CCRCCs) and 7 chromophobe renal cell carcinomas (ChRCCs) based on the carcinoma component. The sarcomatoid components of the investigated tumors resembled these entities as fibrosarcoma, leiomyosarcoma and malignant fibrous histiocytoma (Fig. 1a–d).

The immunohistochemistry results of the two components including carcinoma and sarcomatoid cells were listed in Table 1. The cohorts could be divided into four groups based on the expression panels of each immunohistochemical markers: carcinoma positive and sarcomatoid cell positive (C+/S+); carcinoma positive and sarcomatoid cell negative (C+/S-); carcinoma negative and sarcomatoid cell positive (C−/S+); carcinoma negative and sarcomatoid cell negative (C−/S-). On the whole, the expressions of vimentin, EMA, SMA, and Bcl-2 were significantly different between carcinoma and sarcomatoid cells (*P* < 0.05). No significant difference was revealed in the expressions of other immunohistochemical markers between the two components (*P* > 0.05). The following is the detailed results.

**Table 1:** The immunophenotypes of 42 cases of RCCs with sarcomatoid differentiation and the expression differences between carcinoma and sarcomatoid differentiation

Markers	C+/S+	C+/S-	C−/S+	C−/S-	Total C+	Total S+	*P* value
Vimentin	22	0	20	0	22 (52%)	42 (100%)	0.000*
CK	18	16	3	5	34 (81%)	21 (50%)	0.005*
EMA	21	16	0	5	37 (88%)	21 (50%)	0.000*
CK7	0	11	5	26	11 (26%)	5 (12%)	0.164
CK18	12	13	7	10	25 (60%)	19 (45%)	0.275
HMW-CK	0	0	3	39	0 (0%)	3 (7%)	0.241
CAIX	20	1	12	9	21 (50%)	32 (76%)	0.023*
CD10	20	3	12	7	23 (55%)	32 (76%)	0.065
PAX8	24	10	3	4	34 (81%)	27 (64%)	0.141
AMACR	3	8	3	28	11 (26%)	6 (14%)	0.277
CD117	0	7	3	32	7 (17%)	3 (7%)	0.313
CD99	0	3	9	30	3 (7%)	9 (21%)	0.116
SMA	0	0	8	34	0 (0%)	8 (19%)	0.005*
CD34	0	0	0	42	0 (0%)	0 (0%)	−
S100	0	0	0	42	0 (0%)	0 (0%)	−
HMB45	0	0	0	42	0 (0%)	0 (0%)	−
MelanA	0	0	0	42	0 (0%)	0 (0%)	−
P53	8	2	12	20	10 (24%)	20 (48%)	0.040*
Bcl-2	9	16	0	17	25 (60%)	9 (21%)	0.001*

*C* carcinoma cell, *S* sarcomatoid cell

*The difference is statistically significant

### The expression patterns of vimentin, CK, and EMA in RCCs with sarcomatoid differentiation

The positive expression rates of vimentin were 52% (22/42) in carcinoma cells and 100% (42/42) in sarcomatoid cell (*P* = 0.000). The main expression panels of vimentin were C+/S+ (22/42) and C−/S+ (20/42). The positive expression rates of CK in carcinoma and sarcomatoid cells were 81% (34/42) and 50% (21/42), respectively (*P* = 0.005). The main expression panels of CK were C+/S+ (18/42) and C+/S- (16/42). The positive expression rates of EMA were 88% (37/42) and 50% (21/42) in carcinoma and sarcomatoid cells, respectively (*P* = 0.000). The expression panels of EMA were C+/S+ (21/42) and C+/S- (16/42).

### The expression patterns of CK7, CK18, and HMW-CK in RCC with sarcomatoid differentiation

CK7 was expressed in carcinoma cells in 11 of 42 cases (11/42) and sarcomatoid cells in 5 of 42 cases (5/42) (*P* = 0.164). The expression panels of CK7 were C+/S- (11/42) and C−/S+ (5/42). CK7 was not simultaneously expressed in two types of cells. CK18 was expressed in carcinoma cells in 25 of 42 cases (25/42) and sarcomatoid cells in 19 of 42 cases (19/42) (*P* = 0.275). The main expression panels of CK18 were C+/S+ (12/42) and C+/S- (13/42). HMW-CK was only expressed in the sarcomatoid cells in 3 of 42 cases (3/42) (*P* = 0.241).

### The expression patterns of CAIX, CD10, and PAX8 in RCC with sarcomatoid differentiation

**Fig. 2:**
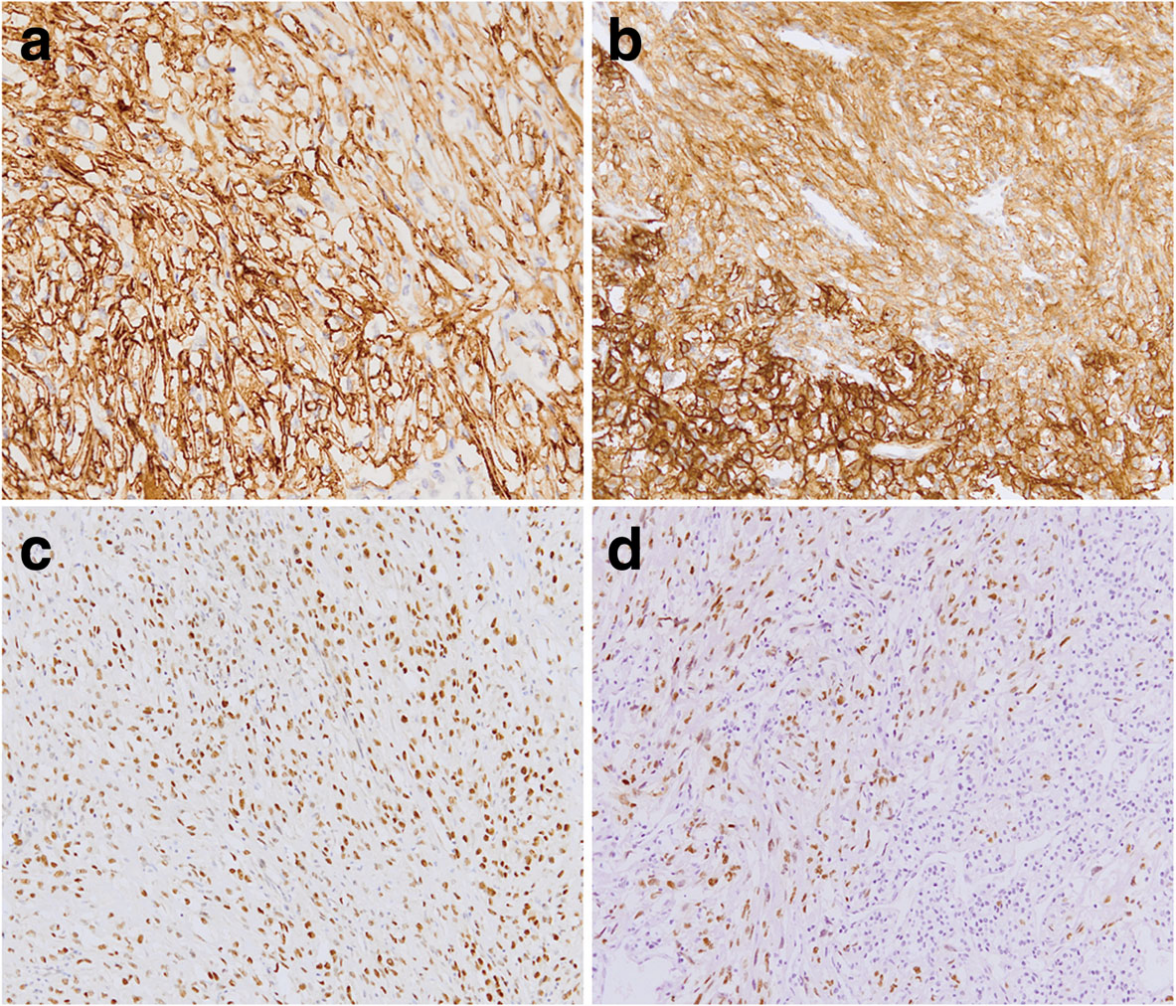
Immunohistochemical staining figures of RCC with sarcomatoid differentiation. **a** The positive expression of CA IX in clear cell carcinoma together with fibrosarcomatoid cells **b** The positive expression of CD10 in clear cell carcinoma together with fibrosarcomatoid cells **c** The positive expression of PAX8 in ChRCC together with fibrosarcomatoid cells **d** The positive expression of p53 in fibrosarcomatoid cells and negative expression in clear cell carcinoma. Immunohistochemistry × 100

The positive expression rates of CAIX in carcinoma and sarcomatoid cells were 50% (21/42) and 76% (32/42), respectively (*P* = 0.023). The major expression panels of CAIXwere C+/S+ (20/42) and C−/S+ (12/42). Among them, CAIX was diffusely strong expressed (3+) in carcinoma cells in 18 of 42 cases and in sarcomatoid cells in 29 of 42 cases. The positive expression rates of CD10 in carcinoma and sarcomatoid cells were 55% (23/42) and 76% (32/42), respectively (*P* = 0.065). The main expression panels of CD10 were C+/S+ (20/42) and C−/S+ (12/42). PAX8 was expressed in carcinoma cells in 34 of 42 cases (34/42) and sarcomatoid cells in 11 of 42 cases (27/42) (*P* = 0.141). Notably, among them, 24 cases showed co-expression of PAX8 in two types of cells (24/42). The main expression panels of PAX8 were C+/S+ (24/42), and C+/S- (10/42). The positive expressions of **CAIX, CD10, and PAX8 were showed in** Fig. 2a, b, and c.

### The expression patterns of AMACR, CD117, and CD99 in RCC with sarcomatoid differentiation

The expression panels of AMACR were C+/S+ (3/42), C+/S- (8/42), and C−/S+ (3/42). The expression patterns showed no difference between carcinoma and sarcomatoid cells (*P* = 0.277). CD117 were merely expressed in carcinoma cells in 7 of 42 cases and sarcomatoid cells in 3 of 42 cases (*P* = 0.313). In addition, 3 samples presented CD99 expression in carcinoma cells and 9 cases presented CD99 expression in sarcomatoid cells (*P* = 0.116).

### The expression patterns of SMA, CD34, S100, HMB45, and melanA in RCC with sarcomatoid differentiation

SMA was expressed in sarcomatoid cells in 8 cases without positive expression in carcinoma cells (*P* = 0.005). But beyond that, CD34, S100, HMB45 and melanA were not expressed in two types of cells in all cases.

### The expression patterns of p53 and Bcl-2 in RCC with sarcomatoid differentiation

Ten cases showed p53 expression in carcinoma cells and 20 cases showed p53 expression in sarcomatoid cells (*P* = 0.04). The main expression panels of p53 were C+/S+ (8/42) and C−/S+ (12/42). The positive expression rates of Bcl-2 in carcinoma and sarcomatoid cells were 60% (25/42) and 21% (9/42), respectively (*P* = 0.001). The expression panels of Bcl-2 were C+/S+ (9/42) and C+/S- (16/42). The positive expression of **p53 were showed in** Fig. 2d.

### Expression differences of CAIX, CD10, vimentin, CK7 and CD117 in the carcinoma and sarcomatoid cells between CCRCC and ChRCC

The expressions of CAIX, CD10, vimentin, CK7 and CD117 in the carcinoma component between CCRCC and ChRCC showed significant differences (*P* < 0.05). However, no significant difference was revealed in the sarcomatoid component between the two tumors (*P* > 0.05) (Table 2).

**Table 2:** The expression differences of CAIX, CD10, vimentin, CK7 and CD117 between CCRCC and ChRCCs in the carcinoma and sarcomatoid differentiation

	CCRCC	ChRCC	*P* value	CCRCC	ChRCC	*P* value
	C+	C+		S+	S+	
CAIX	23/35	0/7	0.002*	29/35	7/7	0.567
CD10	21/35	0/7	0.009*	27/35	5/7	1.000
Vimentin	23/35	0/7	0.002*	35/35	7/7	−
CK7	3/35	7/7	0.000*	5/35	0/7	0.569
CD117	3/35	6/7	0.000*	2/35	0/7	1.000

*C* carcinoma cell, *S* sarcomatoid cell

*The difference is statistically significant

### Clinical follow-up and survival analysis

Follow-up data were collected for 33 cases with the follow-up time ranged from 1 to 42 months. Among them, 1 patient died from other disease rather than tumor. There were another 24 deaths, including 22 CCRCCs and 2 ChRCCs according to the carcinoma elements which occurred in the 1st ~ 30th month after the surgery for metastasis of tumors to the bone or lung. Other 8 patients are still alive uneventfully after surgery.

**Fig. 3:**
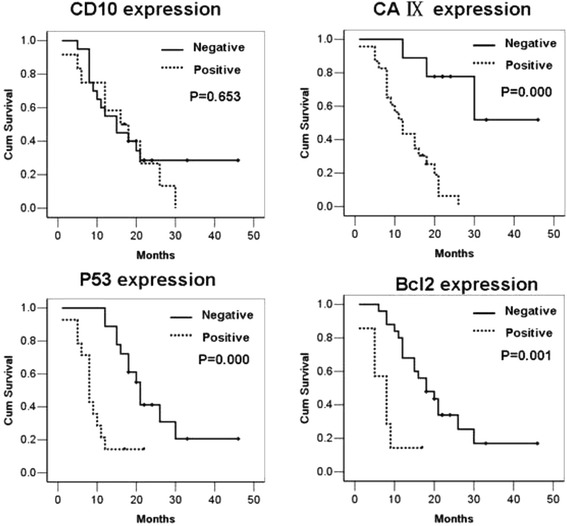
The Kaplan-Meier survival curves comparing overall survival according to the expressions of CD10, CAIX, p53 and Bcl-2. Univariate and multiple analyses showed that the positive expressions of CAIX, p53 and Bcl-2 were all adverse prognostic factors for tumor related survival

The Kaplan-Meier survival curves comparing overall survival according to the expressions of CD10, CAIX, p53 and Bcl-2 were presented in Fig. 3. Univariate analysis showed that the expressions of CAIX (*p* = 0.000), p53 (*p* = 0.000) and Bcl-2 (*p* = 0.001) were all adverse prognostic factors for tumor related survival (Fig. 3). Furthermore, the multiple analyses using the Cox regression model revealed that CAIX, p53 and Bcl-2 were independent predictive factors for prognosis of RCC with sarcomatoid differentiation (*P* = 0.001, 0.011, 0.013, respectively).

## Discussion

A malignant tumor comprising of malignant epithelial cells together with mesenchymal cells is called carcinosarcoma. According to the histological classification of 2016 WHO for the epithelial tumors of kidney, this type of tumor is regarded as transformation from different types of renal cell carcinoma, rather than an independent histological entity [2]. However, a large sample and systematic study on immunophenotypes of both carcinoma and sarcomatoid cells are still limited, especially for some renal specific immunohistochemical markers [4]. Herein we performed immunohistochemistry study to characterize 19 different markers in 42 cases of RCC with sarcomatoid differentiation and analyzed the expression patterns of these markers in both carcinoma and sarcomatoid cells. We grouped these immunohistochemical markers into 4 subgroups as followed based on their different physiological functions.

### Epithelial markers

In the present study, sarcomatoid cells in 81% cases expressed at least one epithelial marker. CK and EMA were expressed in approximately 62% cases. Sarcomatoid cells in 19 cases expressed CK18. However, 8 cases presented no expression of any tested epithelial marker. 5 cases showed CK7 positive staining in sarcomatoid cells rather than in clear cell carcinomas. The expressions of epithelial markers in sarcomatoid cells confirmed that sarcomatoid cells resulted from different differentiation orientations at different degrees of epithelial cells. Thus we recommend a series of cytokeratins with different molecular weights to identify the epithelial expression patterns of sarcomatoid cells.

### Mesenchymal markers

As reported in the literature most sarcomatoid cells expressed vimentin with the positive rates of 56%–100% [5], few cases expressed actin and S100 [6]. In the present study, all 42 cases showed strong positive expression of vimentin in sarcomatoid cells. Among them, 30 cases expressed epithelial markers, 8 cases expressed SMA, but none of them expressed S100. Statistical analysis revealed the expression of vimentin in carcinoma and sarcomatoid cells was significantly different, which might be due to the lack of vimentin expression in 4 ChRCCs. Notably, vimentin was expressed in the sarcomatoid cells of CCRCC, which is different from that of ChRCC.

### RCCs specific markers

CAIX is a valuable marker indicating low endogenous oxygen level, which catalyzes the formation of carbonic acid from carbon dioxide and adjusts the pH value in tumor cells to adapt to the surrounding microenvironment and facilitate tumor proliferation. CAIX is now considered as a highly sensitive and specific marker for clear cell renal cell carcinoma [7]. Immunohistochemical study on CAIX in RCC with sarcomatoid differentiation has been rarely reported up till now. We revealed a high frequency of expression of CAIX in sarcomatoid cells (90%) with extensive and strong staining. Moreover, strong CAIX staining in sarcomatoid cells had been observed regardless the negative expression of CAIX in carcinoma cells (5 CCRCCs, 6 ChRCCs), indicating its involvement in proliferation of sarcomatoid cells and the transformation of carcinoma to sarcomatoid cells. It is known that CD10 could be used as a supplementary diagnostic marker for renal cell carcinoma. 32 cases showed strong CD10 expression in sarcomatoid cells. Among them, CD10 was expressed in both carcinoma and sarcomatoid cells in 20 cases, whereas 12 cases presented CD10 expression only in sarcomatoid cells. PAX8 belongs to PAX gene family, which is an important transcription factor in renal organogenesis and a reliable marker for primary kidney cancer [7]. Chang et al. [8] had reported that the expression rates of PAX8 were 69% and 18% in sarcomatoid cells of RCC with sarcomatoid differentiation and sarcomatoid urothelial carcinoma, respectively. However, it was rarely expressed in renal epithelioid angiomyolipoma and leiomyosarcoma, suggesting that it could be used as a good diagnostic marker for RCC with sarcomatoid differentiation. The present study revealed that PAX8 was expressed in carcinoma cells of 34 cases and sarcomatoid cells of 27 cases. Among them, 24 cases showed extensive expression in both two components, which leads to an idea that PAX8 could be used as a useful diagnostic marker for identifying the RCC with sarcomatoid differentiation in fine needle aspiration cases and in metastatic RCC with sarcomatoid differentiation. AMACR was rarely expressed in sarcomatoid cells, and only 6 cases showed strong AMACR expression in our study.

### Prognosis and treatment related markers

CAIX, one of the most important tumor-associated carbonic anhydrases (CAs), is strongly induced by hypoxia in renal carcinoma cells, which could promote the growth of tumor cells. However, few research endeavors have been devoted to studying its role in predicting the outcome of RCC with sarcomatoid differentiation. Tickoo et al. [9] reported that over expression of CAIX showed no association with survival in 22 clear cell RCCs and 12 nonclear cell RCCs with sarcomatoid differentiation. Whereas, the results of the present study indicated that positive expression of CAIX was related to the adverse prognosis of 42 cases of RCCs with sarcomatoid differentiation. Therefore, more cases are needed for further identifying the predictive function of CAIX for the prognosis of RCCs with sarcomatoid differentiation.

P53 is a tumor suppressor gene, which plays a key role in carcinogenesis. Our study demonstrated that the expression rate of p53 was 48% in sarcomatoid cells, which is higher than that in carcinoma cells, suggesting that p53 might be involved in triggering the development of high malignant sarcomatoid tumors from renal cell carcinoma. Furthermore, p53 could be a reliable marker for predicting the prognosis or treatment outcome of RCC with sarcomatoid differentiation.

Bcl-2 is an apoptosis suppressor gene; therefore its overexpression is a hallmark of cell proliferation and suppression of cell apoptosis. Our result showed a significant difference of Bcl-2 expressions between carcinoma cells and sarcomatoid cells, suggesting that Bcl-2 might facilitate the aberrant growth of carcinoma cells and the transformation of carcinoma cells to high malignant sarcomatoid differentiation via suppression of apoptosis. In addition, the expression of Bcl-2 was associated with the adverse outcome of RCC with sarcomatoid differentiation and Bcl-2 could be an important prognostic factor for the overall survival in RCC with sarcomatoid differentiation.

Additionally, Castillo et al. [10] had reported that sarcomatoid cells in 94.7% of RCC with sarcomatoid differentiation overexpressed CD117, suggesting the idea that tyrosine kinase inhibitor could be used for the therapy of CD117 positive RCC with sarcomatoid components patients. Wang et al. [11] did not find any CD117 expression in sarcomatoid urothelial carcinoma of the upper urinary tract. Our results showed CD117 only expressed in sarcomatoid cells of 3 cases of RCC and carcinoma cells of 7 ChRCCs with sarcomatoid differentiation. More studies are needed to discover the possible therapeutic use of tyrosine kinase inhibitor for RCC with sarcomatoid differentiation.

Due to the rarity of RCCs with sarcomatoid differentiation, more cases are needed to further confirm the specific features of the expression panels of these 19 immunomarkers, especially the prognostic values of CAIX, p53 and Bcl-2 playing in RCCs with sarcomatoid differentiation.

## Conclusions

Sarcomatoid cells in RCC with sarcomatoid differentiation express both epithelial and mesenchymal markers, supporting their epithelial origin. The difference of expression profiles between carcinoma cells and sarcomatoid cells might be due to the distinct orientation and the degree of differentiation in renal cell carcinoma. CAIX, p53 and bcl-2 might play important roles in triggering the transformation from renal cell carcinoma to high malignant sarcomatoid component, and could be beneficial to predicting the prognosis or treatment outcome of RCC with sarcomatoid differentiation. PAX8, CAIX and CD10 could be used as the reliable markers to determine the renal origin of sarcomatoid cells, especially in fine needle aspiration cases and the cases of metastatic RCC with sarcomatoid differentiation. The clinical therapeutic application of tyrosine kinase inhibitors to RCC with sarcomatoid differentiation needs to be investigated with more research studies and clinical trials.

## Abbreviations

AMACR: Alpha-methylacylCoA racemase
CCRCC: Clear cell renal cell carcinoma
ChRCC: Chromophobe renal cell carcinoma
RCC: Renal cell carcinoma
